# Interaction Between the a3 Region of Factor VIII and the TIL’E’ Domains of the von Willebrand Factor

**DOI:** 10.1016/j.bpj.2019.07.007

**Published:** 2019-07-11

**Authors:** Lisbeth Dagil, Kathrin S. Troelsen, Gert Bolt, Lars Thim, Bo Wu, Xin Zhao, Edward G.D. Tuddenham, Thomas E. Nielsen, David A. Tanner, Johan H. Faber, Jens Breinholt, Jakob E. Rasmussen, D. Flemming Hansen

**Affiliations:** 1 Institute of Structural and Molecular Biology, Division of Biosciences, University College London, London, United Kingdom; 2 Novo Nordisk A/S, Novo Nordisk Park, Måløv, Denmark; 3 Department of Chemistry, Technical University of Denmark, Kongens Lyngby, Denmark; 4 Novo Nordisk Research Center China, Beijing, China; 5 Katharine Dormandy, Haemophilia Centre and Thrombosis Unit, Royal Free Hospital NHS Trust, London, United Kingdom

## Abstract

The von Willebrand factor (VWF) and coagulation factor VIII (FVIII) are intricately involved in hemostasis. A tight, noncovalent complex between VWF and FVIII prolongs the half-life of FVIII in plasma, and failure to form this complex leads to rapid clearance of FVIII and bleeding diatheses such as hemophilia A and von Willebrand disease (VWD) type 2N. High-resolution insight into the complex between VWF and FVIII has so far been strikingly lacking. This is particularly the case for the flexible a3 region of FVIII, which is imperative for high-affinity binding. Here, a structural and biophysical characterization of the interaction between VWF and FVIII is presented with focus on two of the domains that have been proven pivotal for mediating the interaction, namely the a3 region of FVIII and the TIL’E’ domains of VWF. Binding between the FVIII a3 region and VWF TIL’E’ was here observed using NMR spectroscopy, where chemical shift changes were localized to two *β*-sheet regions on the edge of TIL’E’ upon FVIII a3 region binding. Isothermal titration calorimetry and NMR spectroscopy were used to characterize the interaction between FVIII and TIL’E’ as well as mutants of TIL’E’, which further highlights the importance of the *β*-sheet region of TIL’E’ for high-affinity binding. Overall, the results presented provide new insight into the role the FVIII a3 region plays for complex formation between VWF and FVIII and the *β*-sheet region of TIL’E’ is shown to be important for FVIII binding. Thus, the results pave the way for further high-resolution insights into this imperative complex.

## Significance

The complex between von Willebrand factor (VWF) and factor VIII (FVIII) is imperative for hemostasis, and failure to form this complex leads to bleeding diatheses such as hemophilia A and von Willebrand disease (VWD). The FVIII a3 acidic region is shown to interact with VWF TIL’E’, and the a3 binding region is localized to two *β*-sheet regions on the periphery of TIL’. Characterizations of the interaction between FVIII and mutants of VWF TIL’E’ further highlight the importance of the *β*-sheet region for binding. The insight into VWF:FVIII complex formation facilitate the design of improved hemophilia A treatments, whereas the analysis of VWD mutations provides a link between genetic pathology and clinical phenotype to facilitate targeted management of patients with VWD.

## Introduction

The interaction between von Willebrand factor (VWF) and coagulation factor VIII (FVIII) is essential to hemostasis. In blood plasma, a tight, noncovalent complex is formed between VWF and FVIII (1, 2), which serves to prolong the half-life of FVIII and locates FVIII to the incipient platelet plug. The interaction between FVIII and VWF is disrupted when FVIII is activated by thrombin; however, if the interaction is intrinsically destabilized, FVIII is rapidly and prematurely cleared from the blood stream (3, 4). This premature clearance of FVIII is caused by a lower affinity of the FVIII:VWF complex and leads to the bleeding disorders hemophilia A and von Willebrand disease (VWD). The severity of these diseases underpins the importance of the interaction between FVIII and VWF and, in turn, the importance of characterizing the structure of this complex at high resolution.

**Figure 1:**
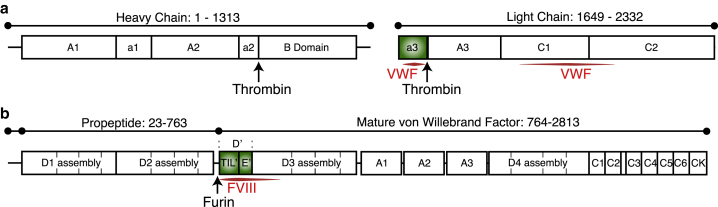
Domain architecture of (*a*) coagulation FVIII and (*b*) the VWF. Specific domains investigated in this study are colored green; that is, the acidic a3 region of FVIII and the TIL’E’ (D’) domains of the VWF. Cleavage sites for thrombin and furin are shown with arrows, and the previously identified binding regions between FVIII and VWF are colored red. To see this figure in color, go online.

Mature FVIII is a 2332 residue protein consisting of six major domains, A1-A2-B-A3-C1-C2, as well as three acidic linker regions a1, a2, and a3 (5) (Fig. 1 *a*). FVIII is divided into a heavy chain (A1-a1-A2-a2-B) and a light chain (a3-A3-C1-C2) (5). The heavy chain and, in particular, the FVIII B-domain are not involved in the interaction with VWF, whereas the FVIII light chain interacts with VWF (1, 6, 7, 8, 9, 10, 11, 12, 13, 14, 15), and the FVIII a3 region has been shown to be important for high-affinity binding (1, 8, 9, 13, 14, 15). The FVIII a3 region spans FVIII residues 1649–1689 and is highly negatively charged. In addition, the FVIII a3 region harbors two of the six posttranslationally sulfated tyrosine residues in FVIII: Tyr1664 and Tyr1680. Whereas sulfation of Tyr1664 is important for activation by thrombin, sulfation of Tyr1680 has been shown to increase the affinity of FVIII for VWF (15). FVIII is activated by thrombin cleavage (16), which results in a heterotrimeric activated FVIII with the FVIII B-domain and a3 region abscized, resulting in an abrogation of the interaction with VWF. Thus, the importance of the FVIII a3 region in mediating the binding with VWF is emphasized by the fact that the complex between FVIII and VWF is disrupted upon thrombin activation and loss of a3 (8).

VWF consists of repeats of homologous domains (17, 18) and is expressed with a propetide that is cleaved off to form mature VWF (Fig. 1 *b*). Mature VWF is a 2050 residue protein starting with the VWF TIL’E’ domains at Ser764 (17). A significant number of binding studies have firmly established the N-terminal part of mature VWF to be the main binding site for FVIII (7, 19, 20, 21, 22). The location of VWD type 2N mutants supports these results, as this VWD subtype is characterized by a quantitative deficiency of FVIII caused by mutations in VWF that impair the interaction with FVIII (23, 24). The majority of VWD 2N mutations are found in the dynamic N-terminal VWF TIL’ domain (see e.g. [25, 26, 27, 28]), with only a single mutation found in the more ordered VWF E’ domain and a minor subset outside the VWF TIL’E’ domains (29, 30). A representation of type 2N mutations on the structure of TIL’E’ recently gave the first structural insight into the major FVIII binding site on VWF and suggested that sites within the TIL’ domain are essential for mediating the high-affinity binding between VWF and FVIII (31).

A recent crystal structure has revealed the VWF TIL’E’D3 assembly at high resolution (32), whereas negative stain electron microscopy (EM) and hydrogen-deuterium exchange mass spectrometry have recently revealed the three-dimensional structure of FVIII in complex with VWF TIL’E’D3 (11, 33). From the structures, it is evident that the VWF TIL’ domain interacts with the FVIII C1 domain, the VWF E’ domain bridges the VWF TIL’ and D3 domains, whereas the VWF D3 domain interacts with the FVIII C1 and C2 domains (33). This picture corresponds well with previously published data showing that binding of an antibody targeting FVIII C1 impairs the binding to VWF (34) and that the FVIII C1 domain is essential for high-affinity interaction with VWF (35). In addition to the structural information deducible from EM, hydrogen-deuterium exchange experiments have shown that the FVIII a3 residues Val1670-Asp1678 are protected upon complex formation with VWF TIL’E’D3, thus indicating that residues in the FVIII a3 region are directly involved in the interaction (11).

The structural insight into the complex between FVIII and VWF presented by the recent three-dimensional structure (11, 33) has provided novel and important information about the VWF:FVIII complex. Although the negative stain EM has contributed to the overall structure of the complex, the nature of the current EM map and in particular its resolution is such that residue-specific interactions cannot be derived and flexible, yet imperative parts such as the FVIII a3 region are absent in the EM map. To understand the interaction between VWF and FVIII at a level where it can be used to guide the developments of improved therapies for patients with hemophilia A or VWD, more detailed information is needed, specifically on the flexible FVIII a3 region and its interaction with VWF.

Using a combination of NMR spectroscopy and isothermal titration calorimetry (ITC), we present a characterization of the interaction between VWF and FVIII with a focus on the interactions mediated by the a3 region. Specifically, we show a direct interaction between the FVIII a3 region and the VWF TIL’ domain and localize the affected residues to two *β*-sheet regions on the VWF TIL’ domain. It is also shown that VWF TIL’E’ binds to full-length FVIII via the TIL’ domain and that this interaction is abrogated when FVIII is activated and the FVIII a3 region is abscized. Finally, mutations introduced in the VWF TIL’E’ domains further highlight the importance of the *β*-sheet regions of TIL’E’ for high-affinity binding.

## Materials and Methods

### Expression and purification of VWF TIL’E’

Four different constructs of VWF TIL’E’ were expressed and purified:

1) A gene coding for an enterokinase site (GTDDDDK) immediately followed by residues 764–865 of VWF was cloned into a pET32b + expression vector with an N-terminal thioredoxin tag followed by a His_6_ tag (GenScript, Hong Kong). This protein was expressed and purified essentially as described in (31), with the exception that the proteolysis step to cleave off the thioredoxin- and His_6_-tag was carried out by adding 1 *μ*L 0.32 *μ*g/mL enterokinase *per* 9 mg protein (New England Biolabs, UK).
2) Expression and purification of the ^15^N-labeled VWF TIL’E’ domains with an N-terminal mutation (GAMG followed by VWF residues 766–864) was carried out as detailed in (31).
3) Expression and purification of VWF TIL’E’ (residues 764–865) with a C-terminal HPC4 purification tag (-ALAEDQVDPRLIDGK) was obtained by preparing a construct with His_6_-DsbC-(G_4_S)_2_-enterokinase site (DDDDK) immediately followed by a gene coding for residues 764–865 of VWF and the C-terminal HPC4 tag (EDQVDPRLIDGK) preceded by a three-residue ALA-linker. This construct was cloned into a pEt-11–derived expression vector and expressed in *Escherichia coli* as detailed in (31). The purification procedure was initiated by sonication followed by homogenization in 20 mM Tris 150 mM NaCl 10 mM imidazole pH 8. The soluble fraction was applied to a NiNTA column and eluted by increasing the concentration of imidazole. This was followed by dialysis into 20 mM Tris 150 mM NaCl 10 mM imidazole pH 8 and subsequent cleavage of the N-terminal tag by addition of enterokinase (1:5000, 4°C overnight). The cleaved fraction was recovered by applying the cleavage reaction to a NiNTA column and collecting the flow-through. The final step of the purification was to apply this to a 30Q anion exchange column and elute TIL’E’-ALA-HPC4 by increasing the NaCl concentration. The correct molecular weight of the protein was verified by mass spectrometry.
4) Unlabeled VWF TIL’E’ was expressed in HKB11 cell line (36, 37). Briefly, TIL’E’-ALA-HPC4 was purified by adding CaCl_2_ (final concentration: 1 mM) to the medium and passing this over an anti-HPC4 column. The protein was eluted from the column using a buffer containing 5 mM EDTA. The identity and purity of the TIL’E’ protein was checked by Edman degradation analysis and sodium dodecyl sulfate-polyacrylamide gel electrophoresis.

For constructs 1, 2, and 3, uniformly ^15^N-labeled samples were obtained by protein overexpression in M9 medium with 1 g/L ^15^NH_4_Cl (U-^15^N labeling) as the sole nitrogen source. For [U-^15^N,^12^C,^2^H; ILV -^13^C^1^H_3_] isotope labeling, the protein was overexpressed in D_2_O based M9 medium with 1 g/L ^15^NH_4_Cl and 3 g/L ^12^C D-Glucose-1,2,3,4,5,6,6-*d*_*7*_. At an optical density at 600 nm of 0.5, the temperature was lowered to 16°C. After 15 min at 16°C, 80 mg/L (2-keto-3-methyl-*d*_*3*_-3-*d*_*1*_-1,2,3,4-^13^C-butyrate) and 60 mg/L (2-keto-3,3-*d*_*2*_-1,2,3,4-^13^C-butyrate) precursors were added. One hour after the addition of the precursors, protein overexpression was induced by the addition of 500 *μ*M isopropyl-*β*-D-1-thiogalactopyranoside.

Due to their identical *K*_d_ for binding to full-length FVIII and their similar ^15^N-^1^H NMR spectra, constructs 1 and 3 were used interchangeably throughout this study and generally referred to as VWF TIL’E’.

### VWD type 2N mutants

The VWD type 2N mutations R854Q and R854K were introduced into the 1 background by standard site-directed mutagenesis. The primers used were 5′-CGGTGCAGTTCCACTTCTGGTCACGGCAAACGCA-3′ and 5′-TGCGTTTGCCGTGACCAGAAGTGGAACTGCACCG-3′. The VWD type 2N mutation R816W was introduced into the 3 background.

### Peptide synthesis

The FVIII a3 peptides and analogs were synthesized by automated solid phase peptide synthesis using the Fmoc/tBu strategy. Peptide syntheses were performed on a 150 *μ*mol scale using Fmoc amino acids. All reactions were run under a nitrogen atmosphere at room temperature. Stock solutions of *N*^*α*^-Fmoc protected amino acid solutions were prepared by dissolving 20 mmol amino acid in 58 mL 0.34 M Oxyma Pure in dimethylformamide (DMF). Sulfated tyrosine residues were incorporated using neopentyl-protected sulfotyrosine (Fmoc-Tyr(SO_3_nP)-OH) and were prepared by dissolving 600 *μ*mol amino acid in 2 mL 0.34 M Oxyma Pure in DMF. A coupling cycle was initiated by Fmoc deprotection with 20% (v/v) piperidine in DMF (2 × 15 min) followed by wash with DMF (9×). Fourfold excess of the amino acid/Oxyma Pure solution was mixed with fourfold excess of *N*,*N*-diisopropylcarbodiimide relative to resin loading, giving a coupling concentration of 0.15 M. The solution was allowed to preactivate for 10 min and was then added to the resin. Coupling proceeded for 1.5 h and was agitated by sparging with nitrogen. The resin was then washed with DMF (4×) and the coupling cycle repeated.

At the end of the synthesis, the resin was washed with DMF (1×) and CH_2_Cl_2_ (6×) followed by drying for 30 min. Finally, the peptides were cleaved from the resin using a cleavage cocktail composed of trifluoroacetic acid, H_2_O, and triisopropylsilane (95:2.5:2.5). Cleaved peptide solutions were concentrated under a stream of nitrogen and precipitated and washed with diethyl ether. The crude neopentyl-protected peptides were dissolved in 2 M ammonium acetate, pH was adjusted to pH 8.5 with NH_4_OH, and the solutions were stirred at 37°C for 8 h. Purification was performed by preparative high performance liquid chromatography on a Phenomenex Gemini-NX C_18_ column using a linear gradient of 5–20% CH_3_CN in 10 mM NH_4_HCO_3_ over 40 min. The purified peptides were lyophilized to yield colorless peptide ammonium salts.

### FVIII

For this study, B-domain deleted FVIII (N8, turoctocog alfa) (38) has been used and is referred to as FVIII throughout. FVIII was activated by addition of thrombin or using the CleanCleave Thrombin kit as detailed by the manufacturer (Sigma-Aldrich, St. Louis, MO).

### NMR spectroscopy

All NMR spectra were recorded at 298 K on Bruker Avance III 700 MHz or Bruker Avance III HD 800 MHz spectrometers equipped with cryoprobes. NMR samples consisted of 10–300 *μ*M isotope labeled TIL’E’ in 10 mM HEPES 300 mM NaCl 5 mM CaCl_2_ 5% D_2_O pH 7.3.

For the characterizations of the interaction between VWF TIL’E’ and FVIII a3 peptides, lyophilized FVIII a3 peptide was dissolved to a final concentration of ∼500 *μ*M directly into an NMR sample already containing 100 *μ*M VWF TIL’E’. pH was subsequently adjusted to 7.3. For the characterizations of the interaction between VWF TIL’E’ and FVIII, VWF TIL’E’ was added to a concentration of 18 *μ*M to a solution of 20 *μ*M FVIII.

Two-dimensional ^15^N-^1^H heteronuclear single quantum correlation (HSQC) spectra (39) were recorded with 3-9-19 Watergate, whereas two-dimensional methyl–transverse relaxation optimized spectroscopy (TROSY) experiments were recorded using the standard heteronuclear multiple quantum coherence pulse scheme (40).

### NMR data analysis

The nmrPipe/nmrDraw program package (41), CCPNmr Analysis version 2.4.2 (42), and FuDA (43) (https://www.ucl.ac.uk/hansen-lab/) were used for processing and analyzing data. Chemical shift differences were calculated as $\Delta \delta =\sqrt{{\left(\Delta \delta_{1\text{H}}\right)}^{2}+{\left(0.15\Delta \delta_{15\text{N}}\right)}^{2}}$.

An upper bound for the dissociation constant, *K*_d_, between VWF TIL’E’ and the FVIII a3 peptides was estimated from the chemical shift changes observed. From the small chemical shift changes observed between spectra of 1) free VWF TIL’E’ and 2) VWF TIL’E’ in the presence of the FVIII a3 peptides, it can be assumed that the binding and dissociation reaction are in the fast-exchange regime (44). In the fast-exchange regime, the observed chemical shift in NMR spectra is given by the population-weighted average of free and bound VWF TIL’E’. The change in chemical shift between free VWF TIL’E’ and TIL’E’ in the presence of FVIII a3 therefore report on the fraction of bound VWF TIL’E’. The possible range of amide ^1^H chemical shifts is between 7 and 9.5 parts per million (ppm), whereas the range on nonglycine ^15^N chemical shifts is between 107 and 132 ppm. The maximal ^1^H shift observed between free and bound TIL’E’ is −0.035 ppm (C821; 8.75 ppm) and the maximal ^15^N shift is +0.36 ppm (N819; 114.5 ppm), both in the presence of a3_sTyr1664-sTyr1680_. This leads to a population of the bound state of VWF TIL’E’ of *p*_bound_ > 0.02. In the experiments, the concentration of TIL’E’ was 100 *μ*M and the concentration of FVIII a3_sTyr1664-sTyr1680_ was 500 *μ*M, thereby leading to a *K*_d_ < (0.98 × 100 × 498 *μ*M)/(0.02 × 100 *μ*M) = 24.4 mM ≈ 25 mM.

### ITC

ITC experiments were performed on a MicroCal iTC200 (Malvern Instruments, Malvern, UK) at 25°C. All experiments were performed with a total of 19 injections (the first injection 0.4 *μ*L, the rest 2 *μ*L), with 120 s between each injection and an initial delay of 60 s. The reference power was set to 6 *μ*cal/s, and the syringe was stirred at 750 or 1000 Rpm depending on the specifications of the syringe. The concentrations of proteins used are specified in the figure legends.

## Results and Discussions

### Interaction between VWF TIL’E’ and FVIII a3

The acidic a3 region of FVIII (Fig. 1 *a*) is known to be essential for mediating the high-affinity interaction between VWF and FVIII (1, 8, 9, 13, 14, 15); however, the mechanism by which the FVIII a3 region mediates this interaction is unknown. With the structure and NMR chemical shift assignment of the VWF TIL’E’ domains (Fig. 1 *b*) now available (31), it becomes possible to characterize possible direct interactions between FVIII a3 and VWF TIL’E’ and therefore possible to probe the mechanism by which the a3 region mediates the interaction between FVIII and VWF.

Peptides representing the FVIII a3 region were used to probe the interaction between FVIII a3 and VWF TIL’E’. These peptides can be produced in relatively high amounts and to a high homogeneity. Peptides can also be produced to mimic different sulfation patterns of FVIII, thereby probing the importance of Tyr1680 sulfation in FVIII a3 for the interaction with VWF. Four FVIII a3 peptides spanning residues 1649–1689 of FVIII were synthesized with different sulfation patterns on Tyr1664 and Tyr1680: a3_nonsulf_ without any sulfations, a3_sTyr1664_ with Tyr1664 sulfated, a3_sTyr1680_ with Tyr1680 sulfated, and a3_sTyr1664-sTyr1680_ with both Tyr1664 and Tyr1680 sulfated.

**Figure 2:**
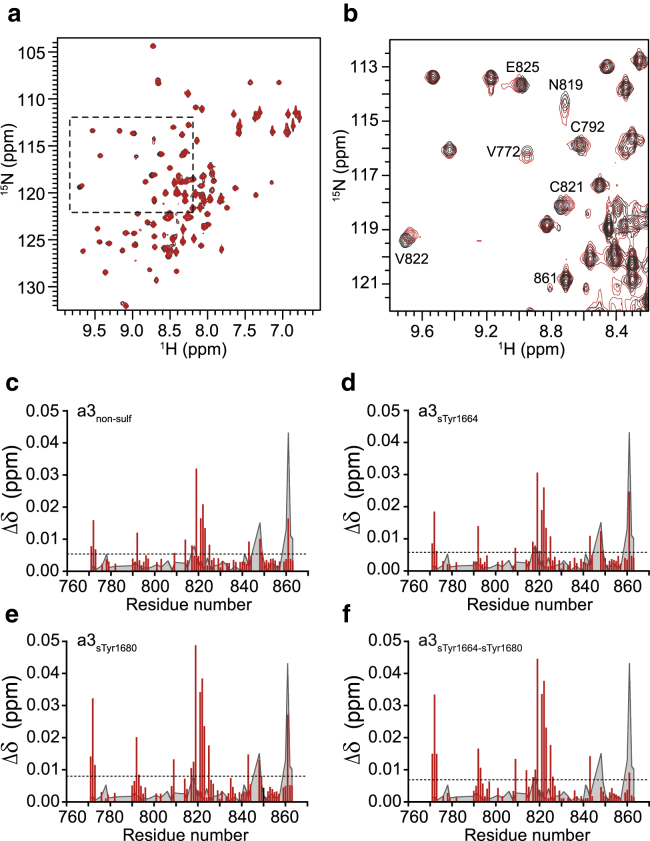
NMR spectrum showing the interaction between VWF TIL’E’ and FVIII a3. (*a*) Overlay of ^15^N-^1^H HSQC spectra of free VWF TIL’E’ (*black*) and VWF TIL’E’ (100 *μ*M) in the presence of five molar equivalent a3_sTyr1664-sTyr1680_ (500 *μ*M) (*red*). (*b*) Zoom of the boxed area in (*a*) where cross peaks with significant chemical shift changes are annotated. Both spectra were recorded at a temperature of 298 K. (*c*)–(*f*) Chemical shift changes, *Δδ*, observed in VWF TIL’E’ upon addition of five molar excess of differently sulfated a3 peptides, (*c*) a3_nonsulf_, (*d*) a3_sTyr1664_, (*e*) a3_sTyr1680_, and (*f*) a3_sTyr1664-sTyr1680_. The dashed line shows the average chemical shift changes in the individual experiments and therefore indicates the confidence level. The gray shaded graphs in the background are the chemical shift changes observed for VWF TIL’E’ between pH 7.0 and 7.5. Any chemical shift changes that are smaller than the pH-induced chemical shift changes are not considered significant. A primary binding region around residue 820 is observed and secondary binding regions around residues 770 and 795. To see this figure in color, go online.

NMR spectroscopy is well-suited to characterize low-affinity protein complexes with dissociation constants, *K*_d_, up to the high millimolar range (45). Initial ITC experiments, Fig. S1, showed that a possible interaction between FVIII a3 and VWF TIL’E’ would be in the millimolar range. The interaction between VWF TIL’E’ and the four FVIII a3 peptides was therefore probed using NMR spectroscopy. NMR samples of uniformly ^15^N-labeled VWF TIL’E’ with unlabeled FVIII a3 peptide in approximately five times molar excess (see Materials and Methods) were prepared and ^15^N-^1^H HSQC spectra were recorded and compared with spectra of free VWF TIL’E’. Fig. 2, *a* and *b* show overlays of HSQC spectra of free VWF TIL’E’ and VWF TIL’E’ in the presence of FVIII a3_sTyr1680_. In Fig. 2, *c*–*f*, the chemical shift changes, *Δδ* = $\sqrt{{\left(\Delta \delta_{\text{H}}\right)}^{2}+{\left(0.15\;\Delta \delta_{\text{N}}\right)}^{2}}$, between free VWF TIL’E’ and VWF TIL’E’ in the presence of the FVIII a3 peptides are shown. As seen in Fig. 2, the chemical shift changes are in general small with *Δδ* < 0.01 ppm for most of the residues. Residues in VWF TIL’E’ with *Δδ* > 0.01 ppm include Met771, Val772, Lys773, Cys792, Gln793, Leu809, Glu818, Asn819, Cys821, Val822, Ala823, Asn825, and Lys843, and the highest chemical shift changes (*Δδ* > 0.03 ppm) are observed for Val772, Asn819, Cys821, and Val822 all located in the VWF TIL’ subdomain.

**Figure 3:**
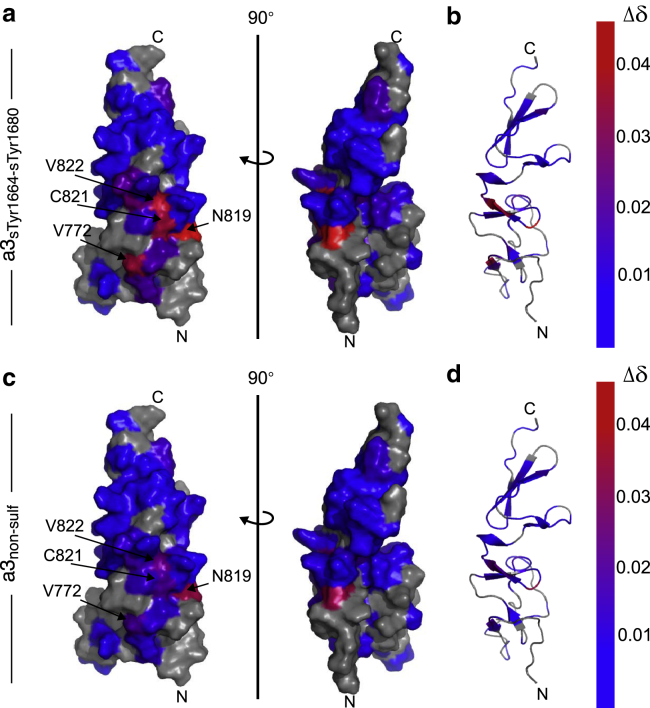
Chemical shift changes observed upon FVIII a3 peptide-binding shown on the structure of VWF TIL’E’ (Protein Data Bank [PDB]: 2MHP). (*a*) Chemical shift changes, *Δδ*, upon addition of five molar excess of FVIII a3_sTyr1664-sTyr1680_ to VWF TIL’E’. Red colors show the residues with the largest chemical shift changes, blue smallest changes, and gray show residues where no data are available. (*b*) Chemical shift changes shown on the secondary structure elements of VWF TIL’E’ localize these changes to the periphery of the two *β*-sheets in the VWF TIL’ subdomain. (*c*) Chemical shift changes, *Δδ*, upon addition of five molar excess of FVIII a3_nonsulf_ to VWF TIL’E’. (*d*) Chemical shift changes from (*c*) shown on the secondary structure elements of VWF TIL’E’. Residues for which the chemical shift changes caused by a3 binding are smaller than the pH-induced shift are colored blue. To see this figure in color, go online.

The majority of the peaks overlay well, thus indicating that there are no major structural changes in VWF TIL’E’ upon interaction with FVIII a3. It is interesting to note that a subset of peaks show significant chemical shift changes. A change in chemical shifts in a structurally defined region upon addition of a binding partner reports on the site of interaction (46). These chemical shift changes can originate from small conformational changes or from electrostatics or ring-current effects. When the chemical shift changes observed in Fig. 2 are mapped on the structure of VWF TIL’E, they highlight two regions. For the residues with Δδ>0.03ppm, Val772 is located on the edge of the *β*1-*β*2 sheet, and Asn819, Cys821, and Val822 are located on the *β*4 strand (Fig. 3).

Because the chemical shift changes are generally small, separate experiments were devised to verify that these shifts are not simply due to small changes in the sample conditions, such as pH and concentration of salts. Specifically, a pH titration from pH 6.5 to 8 in steps of 0.5 pH unit and a titration of the NaCl concentration from 100 to 250 mM in steps of 50 mM were performed (Fig. S2). Significant shifts were only observed in the pH titration, whereas no significant shifts were observed in the titration with NaCl. The chemical shift changes observed in the pH titration are taken into account in the interpretation of the data (see Figs. 2 and 3). Thus, chemical shift changes that are smaller than the pH-induced shift are not considered significant.

It is important to state that saturation could not be reached in any of the titrations, and it is therefore not possible to obtain an accurate value for the *K*_d_ between FVIII a3 and VWF TIL’E’ from the NMR data. However, from the chemical shift changes obtained, e.g. up to 0.035 ppm in the ^1^H dimension and from the range of possible amide ^1^H chemical shifts (7–9.5 ppm), an upper bound for the *K*_d_ can be estimated to *K*_d_ ≲ 25 mM (see Materials and Methods).

Interestingly, the addition of FVIII a3 peptides with Tyr1680 sulfated (a3_sTyr1664-sTyr1680_ and a3_sTyr1680_) give significantly larger chemical shift changes compared to FVIII a3 peptides where Tyr1680 is not sulfated (a3_nonsulf_ and a3_sTyr1664_). The residue-specific chemical shift changes caused by the different FVIII a3 peptides are proportional, thereby confirming that the chemical shift changes are due to protein-peptide interactions (Fig. S3). The residue-specific chemical shift changes increase by a factor of 1.66 ± 0.09 when Tyr1680 is sulfated (Fig. S3), in accord with previous accounts, showing that Tyr1680 sulfation increases the binding affinity (15). In contrast, there is no significant increase in the chemical shift changes when Tyr1664 is sulfated (a factor of 1.03 ± 0.05).

As a further control for the interaction between FVIII a3 and VWF TIL’E’, the interaction between a severe VWD type 2N mutation of VWF (R816W) and FVIII a3_sTyr1664-sTyr1680_, was characterized. Arg816 is located in the VWF TIL’ *β*3-*β*4 sheet (31), where the largest chemical shift changes were observed (Figs. 2 and 3). Also, it has previously been shown that full-length VWF R816W does not bind FVIII in enzyme-linked immunosorbent assays (25). To assess this, ^15^N-^1^H HSQC spectra of free VWF TIL’E’ R816W and VWF TIL’E’ R816W in the presence of five molar excess of FVIII a3_sTyr1664-sTyr1680_ were recorded, similar to the experiments in Fig. 2. A subset of peaks within VWF TIL’E’ R816W was slightly affected upon addition of FVIII a3_sTyr1664-sTyr1680_ (Fig. S4). However, the most affected residues are located far from each other on the three-dimensional structure of VWF TIL’E’. In addition, the chemical shift changes observed for VWF TIL’E’ R816W are substantially smaller than those observed for wild-type VWF TIL’E’ in the presence of FVIII a3_sTyr1664-sTyr1680_, thereby substantiating that the *β*3- *β*4 sheet is important for interaction and that the R816W mutation decreases the affinity between FVIII a3 and VWF TIL’E’.

### Interaction between FVIII and VWF TIL’E’

**Figure 4:**
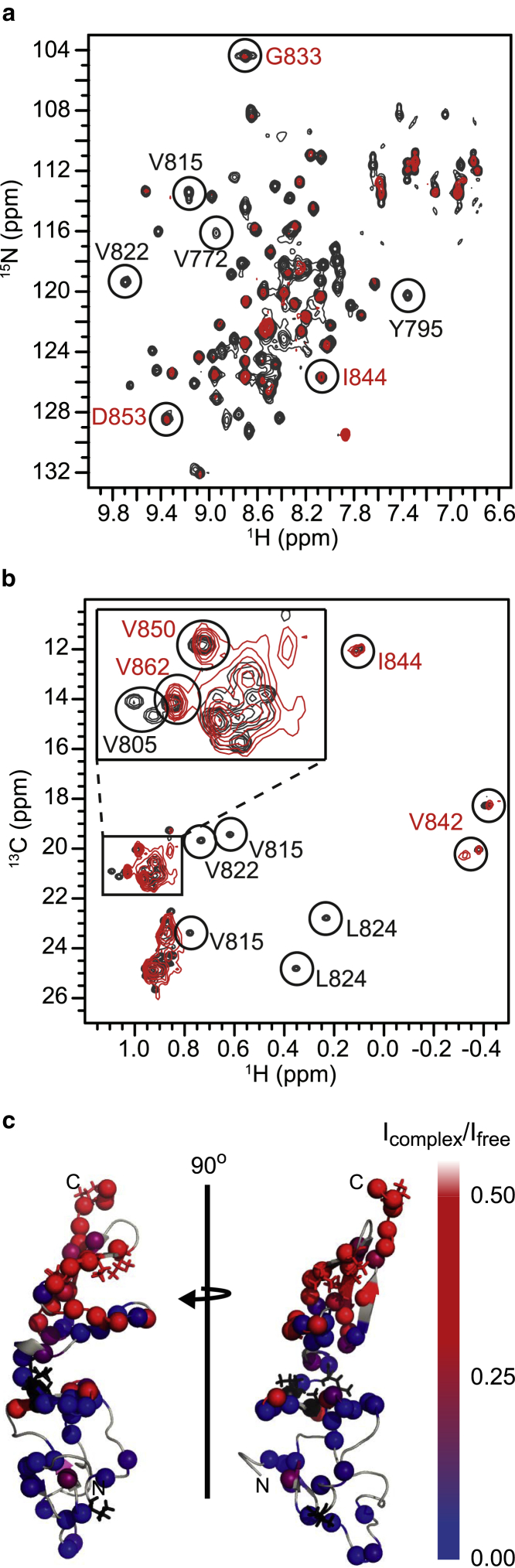
NMR spectra of VWF TIL’E’ in the presence of FVIII localize the interaction site to the N-terminal VWF TIL’ domain. (*a*) ^15^N-^1^H NMR correlation spectrum showing free VWF TIL’E’ (*black*) and VWF TIL’E’ (18 *μ*M) in the presence of molar excess (20 *μ*M) of FVIII (*red*). (*b*) ^13^C-^1^H NMR methyl-TROSY correlation spectrum showing free VWF TIL’E’ (*black*) and VWF TIL’E’ (18 *μ*M) in the presence of molar excess (20 *μ*M) of FVIII (*red*). For both (*a*) and (*b*), a subset of the cross peaks that disappear upon complex formation with FVIII are encircled and denoted in black writing, whereas a subset of the cross peaks that are visible in both free and complexed form of VWF TIL’E’ are encircled and denoted in red writing. (*c*) Residue-specific intensity ratios, *I*_complex_/*I*_free_, colored on the structure of VWF TIL’E’ (PDB: 2MHP). The intensity ratios are calculated from the intensity of the respective cross-peak in the ^15^N-^1^H spectrum of free VWF TIL’E’ (*I*_free_) and the cross peaks in the ^15^N-^1^H spectrum of VWF TIL’E’ in the presence of molar excess of FVIII (*I*_complex_). Side chains circled in (*b*) are shown as sticks colored black for peaks that disappear and red for peaks that are visible in the ^13^C-^1^H spectrum. To see this figure in color, go online.

Binding experiments with FVIII, as opposed to just the a3 region, were performed to further characterize the interaction between VWF and FVIII. The addition of substoichiometric amounts (1:3) of FVIII to a sample of ^15^N-labeled VWF TIL’E’ did not lead to chemical shift changes of the VWF TIL’E’ cross peaks in ^15^N-^1^H HSQC spectra; instead, it led to a change in the peak intensities vide infra. This observation shows that the exchange between bound and free TIL’E’ is in the intermediate to slow-exchange regime (44, 47), meaning that the dissociation rate of the TIL’E’:FVIII complex is slower than the chemical shift differences (rad/s) between the peaks of free and bound VWF TIL’E’. The effect of binding was therefore characterized using two samples, a sample with “free” ^15^N-labeled VWF TIL’E’ and a sample with “bound” VWF TIL’E’ made by adding a slight molar excess of unlabeled FVIII. A ^15^N-^1^H HSQC spectrum of bound VWF TIL’E’ is compared to free VWF TIL’E’ in Fig. 4 *a*.

A total of 94 peaks are theoretically possible for the VWF TIL’E’ construct in ^15^N-^1^H HSQC spectra; however, in the spectrum of the VWF TIL’E’:FVIII complex, only ∼30 peaks are observed. The VWF TIL’E’ peaks observed in the ^15^N-^1^H HSQC spectrum of the complex originate primarily from the VWF E’ domain, and the peak intensities vary substantially over the structure (Fig. 4 *c*), with the peaks from residues in VWF TIL’ being most reduced. Reduced intensity of peaks in the spectrum is the result of line-broadening caused by the large size of the complex (∼180 kDa) and chemical exchange between a bound and unbound state, albeit on a timescale that is not suited for NMR spectroscopy. To further characterize the interaction, an alternative labeling strategy was employed. NMR spectroscopy of proteins with selectively isotope labeled ^13^C^1^H_3_-methyl groups in a completely deuterated background has emerged as an eminent technique to probe large macromolecular complexes due to the favorable relaxation properties of the methyl groups (48, 49). Therefore, specific ^13^C^1^H_3_-labeling of isoleucine ^13^C^*δ*1^, valine ^13^C^*γ*1^, ^13^C^*γ*2^, and leucine ^13^C^*δ*1^, ^13^C^*δ*2^ of VWF TIL’E’ was employed. VWF TIL’E’ has a total of 16 Ile, Leu, and Val residues evenly distributed over the structure, theoretically giving rise to 31 methyl peaks in a ^13^C-^1^H methyl-TROSY experiment (50). In the spectrum of ^13^C^1^H_3_ Ile, Leu, and Val labeled VWF TIL’E’ 29 peaks are distinguishable (Fig. 4 *b*). The chemical shifts of these peaks have previously been stereospecifically assigned (31). When adding a slight molar excess of FVIII to VWF TIL’E’, the majority of the methyl peaks in the N-terminal VWF TIL’ domain disappears. This supports the results obtained above, showing that primarily VWF TIL’ is affected by the interaction with FVIII. Even though this labeling scheme is optimal for large proteins and complexes, the method is dependent on per-deuteration of all sites near the observed methyl groups. FVIII is produced in mammalian cell lines and as such a fully deuterated sample of FVIII could not be obtained. Many of the methyl groups of Leu and Val residues in VWF TIL’ are surface exposed, and the disappearance of peaks in the methyl-TROSY spectra is therefore most likely caused by the rapid relaxation caused by the ^1^H in FVIII. The low concentration of the sample precluded a more quantitative characterization of the binding kinetics. Despite these difficulties, this experiment still underlines that the region most involved in complex formation with FVIII is VWF TIL’ in agreement with the titration of FVIII a3 shown above (Fig. 3 *c*).

### Perturbing the interaction between FVIII and VWF TIL’E’

VWD type 2N mutations are already known to cause structural perturbations of VWF (51) that lead to a reduced VWF:FVIII binding affinity, and mapping these mutations onto the three-dimensional structure of VWF TIL’E’ has already provided insight into the FVIII binding region on VWF (31). From the experiments above, it is clear that the N-terminal VWF TIL’ subdomain and the FVIII a3 region play important roles for the interactions between VWF and FVIII. To characterize the interaction between FVIII and VWF TIL’E’ further, FVIII was activated by thrombin, during which the FVIII a3 region is cleaved off. As seen in Fig. S5, the spectrum of VWF TIL’E’ in the presence of FVIII, but upon thrombin activation of FVIII and production of activated FVIII, is identical to the spectrum of free VWF TIL’E’. Therefore, activation of FVIII and cleaving off FVIII a3 leads to a dissociation of the complex between FVIII and VWF. Thus, as shown previously (8), the FVIII a3 region is important for high-affinity binding between VWF TIL’E’ and FVIII. Furthermore, this experiment supports that the disappearance of VWF TIL’ peaks in the NMR spectrum of the VWF TIL’E’:FVIII complex is caused by an interaction between FVIII and VWF TIL’E’.

**Figure 5:**
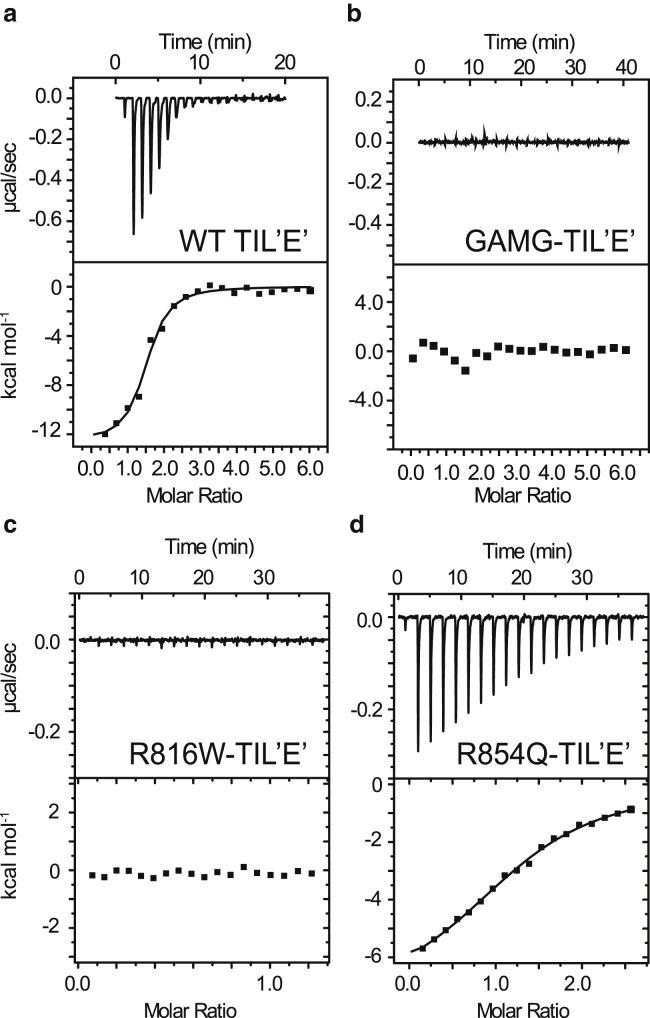
ITC experiment showing binding between VWF TIL’E’ and FVIII. (*a*) Representative ITC experiment showing the binding between VWF TIL’E’ and FVIII. Three independent ITC experiments were used to obtain the thermodynamic parameters and their uncertainties: *K*_d_ = 0.71 ± 0.26 *μ*M, *N* = 1.0 ± 0.3, ΔH=−12.0 ± 1.3 kcal/mol. (*b*) ITC experiment showing substantially reduced binding between the VWF TIL’E’ construct with the additional N-terminal glycine and alanine residues, VWF GAMG-TIL’E’, and FVIII. (*c*) Substantially reduced binding between VWF R816W TIL’E’ and FVIII. (*d*) Weak binding between VWF R854Q TIL’E’ and FVIII: *K*_d_ = 7.2 ± 0.6 *μ*M, *N* = 1.29 ± 0.03, ΔH=−7.5 ± 0.2 kcal/mol (one run).

The dissociation constant for the VWF TIL’E’:FVIII complex is 0.7 ± 0.3 *μ*M from ITC experiments (Fig. 5 *a*), whereas the dissociation constant for the VWF TIL’E’:FVIII a3 complex was estimated above to be <25 mM. The FVIII a3 region therefore contributes about one-third of the binding free energy for the formation of the VWF TIL’E’:FVIII complex. Dissociation of the VWF TIL’E’:FVIII complex upon activation by thrombin is in agreement with the determined dissociation constants. It should be noted, however, that the FVIII a3 region could sample different conformations in isolation and covalently bond to the FVIII A3 domain, which could alter its contribution to the binding energy.

Mutations were made in the VWF TIL’E’ regions that showed chemical shift changes upon interaction with FVIII a3. Chemical shift changes are observed in the *β*1-*β*2 region upon FVIII a3 binding to VWF TIL’E’ and previous studies have emphasized the importance of the native N-terminus of mature VWF in mediating the binding to FVIII. These include that the addition of an N-terminal alanine residue abolishes binding (52, 53). A construct of VWF GAMG-TIL’E’, corresponding to the four amino acids GAMG followed by residues 766–864 of VWF, TIL’ was conveniently available to this study and expresses well in *E. coli*. Importantly, the GAMG-TIL’E’ construct has an additional alanine residue in position 763, which was previously shown to abolish binding to FVIII, as well as a glycine in position 762. An ITC experiment on the VWF GAMG-TIL’E’ construct confirmed that the binding to FVIII had been abrogated (Fig. 5, *a* and *b*) by the additional amino acids. The VWF GAMG-TIL’E’ construct was further characterized using two-dimensional ^15^N-^1^H HSQC spectra to deduce the origin of the decrease in binding affinity. Specifically, a comparison of the spectrum of the wild-type construct, VWF TIL’E’, and the VWF GAMG-TIL’E’ construct with the additional alanine residue, showed very few differences in chemical shifts and peak intensities (Fig. S6). Three residues had chemical shift differences significantly larger than the mean chemical shift difference (〈*Δδ* 〉) of 0.04 ppm; these were Lys773, Cys808, and Leu809 (〈*Δδ* 〉 0.06–0.12 ppm). As such, the structural and dynamical differences caused by the additional N-terminal glycine and alanine residues are minor; yet, the *β*1-*β*2 sheet is perturbed. Consequently, the lower binding affinity of VWF GAMG-TIL’E’ is not due to overall structural perturbations but is most likely caused by a local perturbation to the *β*1-*β*2 region. Two control constructs were produced. The first construct had 15 additional C-terminal residues (-ALAEDQVDPRLIDGK), corresponding to a HPC4 purification tag that was not cleaved off, which did not affect the binding between VWF TIL’E’ and FVIII (*K*_d_ = 0.64 ± 0.16 *μ*M). The second control construct was expressed in a mammalian cell line, which also did not affect the binding (*K*_d_ = 0.75 ± 0.25 *μ*M) and resulted in an ^15^N-^1^H HSQC spectrum similar to VWF TIL’E’ produced in bacterial cells (Fig. S7). Of the peaks present, only small chemical shift changes were observed between VWF TIL’E’ produced in bacterial cells and VWF TIL’E’ produced in mammalian cells. The good agreement between peak position and relative peak intensity of the two spectra show that correct folding and disulfide bridge formation is achieved from bacterial *E. coli* expression.

**Figure 6:**
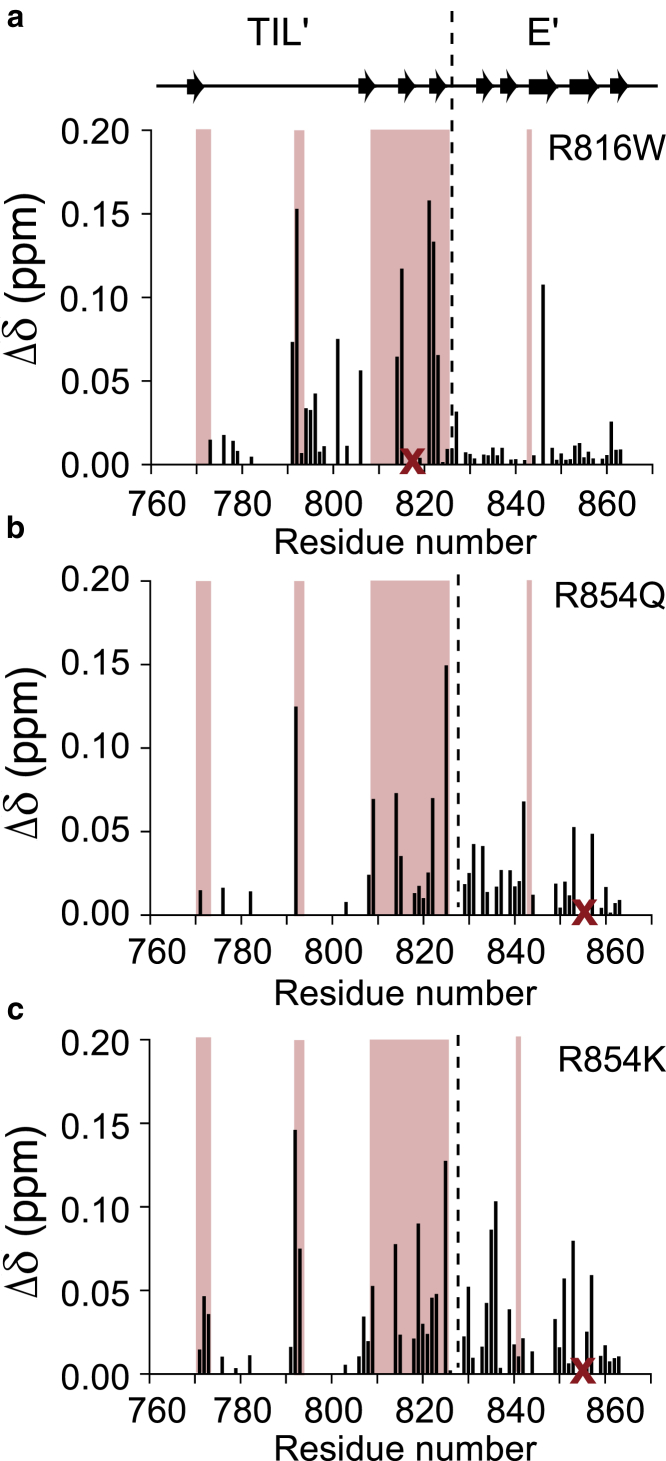
Mapping chemical shift changes between wild-type VWF TIL’E’ and the type 2N mutants R816W and R854Q as well as R854K. (*a*) Chemical shift changes, *Δδ*, between wild-type and R816W. (*b*) Chemical shift changes, *Δδ*, between wild-type and R854Q. (*c*) Chemical shift changes, *Δδ*, between wild-type and R854K. The secondary structure elements are shown above the plot and the dashed line shows the border between the VWF TIL’ and E’ domains. The areas significantly affected by the interaction with FVIII a3_sTyr1664-sTyr1680_ are shaded red. The red X denotes the site of mutation in each panel. To see this figure in color, go online.

Mutations in the *β*3-*β*4 sheet region of VWF TIL’E’ are already known to abrogate VWF:FVIII binding. For example, the severe type 2N mutation R816W is located in the VWF TIL’ *β*3-*β*4 sheet (31). As expected, no binding was observed between FVIII and VWF TIL’E R816W in ITC experiments (Fig. 5 *c*). To further characterize this mutant and the cause of its impaired FVIII binding, a ^15^N-^1^H HSQC spectrum was recorded, and the chemical shift changes between VWF R816W TIL’E’ and wild-type VWF TIL’E’ were quantified (Fig. 6 *a*). The effect of introducing the R816W mutations is observed near the site of the mutation in the VWF TIL’ domain (Fig. 6 *a*), in the *β*3-*β*4 *β*-sheet (residues 814–824), and in the region where VWF TIL’ and E’ interacts (residues 792–795).

The type 2N mutant R854Q, which leads to a mild phenotype VWD, is located in the E’ domain, and it was previously not clear how this mutation affects the VWF:FVIII binding. The R854Q mutation leads to an increase in *K*_d_ (*K*_d_ = 7.2 ± 0.6 *μ*M) by about an order of magnitude compared to the wild-type (Fig. 5 *c*), and R854K leads to a more than 20-fold increase in *K*_d_ (*K*_d_ = 25 ± 20 *μ*M) as shown by ITC experiments. As above, two-dimensional ^15^N-^1^H HSQC spectra and chemical shift changes between VWF R854Q/K TIL’E’ and wild-type VWF TIL’E’ were used to probe a possible mechanism. Although the R854Q and R854K mutations are located in the VWF E’ domain, both of these mutations lead to significant chemical shift changes in the VWF TIL’ domain, and, importantly, the chemical shift changes coincide with the regions that are affected by FVIII a3 binding (Fig. 6, *b* and *c*). One possible route by which the R854Q/K mutations decrease the affinity of TIL’E’ for FVIII is therefore due to an allosteric perturbation of the *β*1-*β*2 and *β*3-*β*4 *β*-sheets of the VWF TIL’ domain. In that regard, it is interesting to note that the R854K mutation, which preserves charge, leads to a larger (allosteric) perturbation in the *β*-sheets of the VWF TIL’ domain and a weaker FVIII binding affinity.

## Conclusions

It has previously been suggested that the FVIII a3 region is responsible for mediating high-affinity binding to VWF, and although it was known that the tyrosine sulfation pattern of the FVIII a3 region affects the VWF:FVIII binding affinity, the mechanism by which the FVIII a3 region stabilizes the VWF:FVIII interaction was not known. Significant chemical shift changes in NMR spectra of VWF TIL’E’ were observed upon addition of FVIII a3 peptides, which shows a direct interaction between VWF TIL’E’ and the FVIII a3 region, albeit with millimolar affinity. The chemical shift changes are observed near the two *β*-sheets of the VWF TIL’ subdomain (*β*1-*β*2 and *β*3-*β*4). The micromolar interaction between VWF TIL’E’ and FVIII was further characterized using NMR and ITC. Firstly, activated FVIII that lacks the a3 region does not interact with VWF TIL’E’. Secondly, mutations to VWF TIL’E’ that perturb the two *β*-sheets in VWF TIL’, *β*1-*β*2 and *β*3-*β*4, destabilize the interaction between VWF TIL’E’ and FVIII. Specifically, a construct with just two additional N-terminal amino acids, VWF GAMG-TIL’E’, leads to chemical shift changes in the *β*1-*β*2 region, and this construct is unable to bind FVIII with micromolar affinity. Additional mutants of VWF TIL’E’ that have a lower affinity for FVIII also show chemical shift changes in the two *β*-sheets. Two of these mutants (R854K/Q) are distant to the *β*1-*β*2 and *β*3-*β*4 *β*-sheets, but the mutations still lead to chemical shift changes in the region where FVIII a3 interacts.

Characterizing the complex between VWF and FVIII is crucial for deducing the molecular functions of these proteins to increase our understanding of the molecular basis for bleeding diatheses, such as VWD and hemophilia A. The complex between VWF and FVIII has therefore attracted substantial attention over the last decades (7, 19, 20, 21, 22). The recent three-dimensional structures of the VWF TIL’E’ domains (31), the TIL’E’D3 module (32), and the FVIII:TIL’E’D3 complex (11, 33) have provided the necessary platform to interpret the findings above in a structural context. The localization of the binding site of the FVIII a3 on VWF TIL’E’ to the *β*-sheet region of VWF TIL’ provides an important step toward a high-resolution structural characterization of the complex between VWF and FVIII and a detailed molecular understanding of this essential complex in biology and in bleeding disorders.

## Author Contributions

L.D. produced the VWF TIL’E’ constructs 1 and 3 and conducted NMR and ITC experiments. K.S.T. and J.E.R. designed and produced the FVIII a3 peptides. G.B. and L.T. produced and purified the VWF TIL’E’ from HKB11 and recombinant FVIII. B.W. and X.Z. produced VWF TIL’E’ with the C-terminal HPC4 tag. J.B., E.G.D.T., J.H.F., and D.F.H. supervised and designed the research. L.D., J.B., J.H.F., J.E.R., and D.F.H. analyzed the data and wrote the article.

## Supporting Material

Document S1. Figs. S1–S7

Document S2. Article plus Supporting Material
