# Supplementary material for: Interaction Between the a3 Region of Factor VIII and the TIL’E’ Domains of the von Willebrand Factor
Source: Biophys J. 2019 Jul 11;117(3):479–89. doi: 10.1016/j.bpj.2019.07.007 (PMC6697466; doi:10.1016/j.bpj.2019.07.007)
Supplement: Document S1. Figs. S1–S7 [file mmc1.pdf]

Biophysical Journal, Volume *117*

Supplemental Information

Interaction Between the a3 Region of Factor VIII and the TIL'E' Domains of the von Willebrand Factor

Lisbeth Dagil, Kathrin S. Troelsen, Gert Bolt, Lars Thim, Bo Wu, Xin Zhao, Edward G.D. Tuddenham, Thomas E. Nielsen, David A. Tanner, Johan H. Faber, Jens Breinholt, Jakob E. Rasmussen, and D. Flemming Hansen

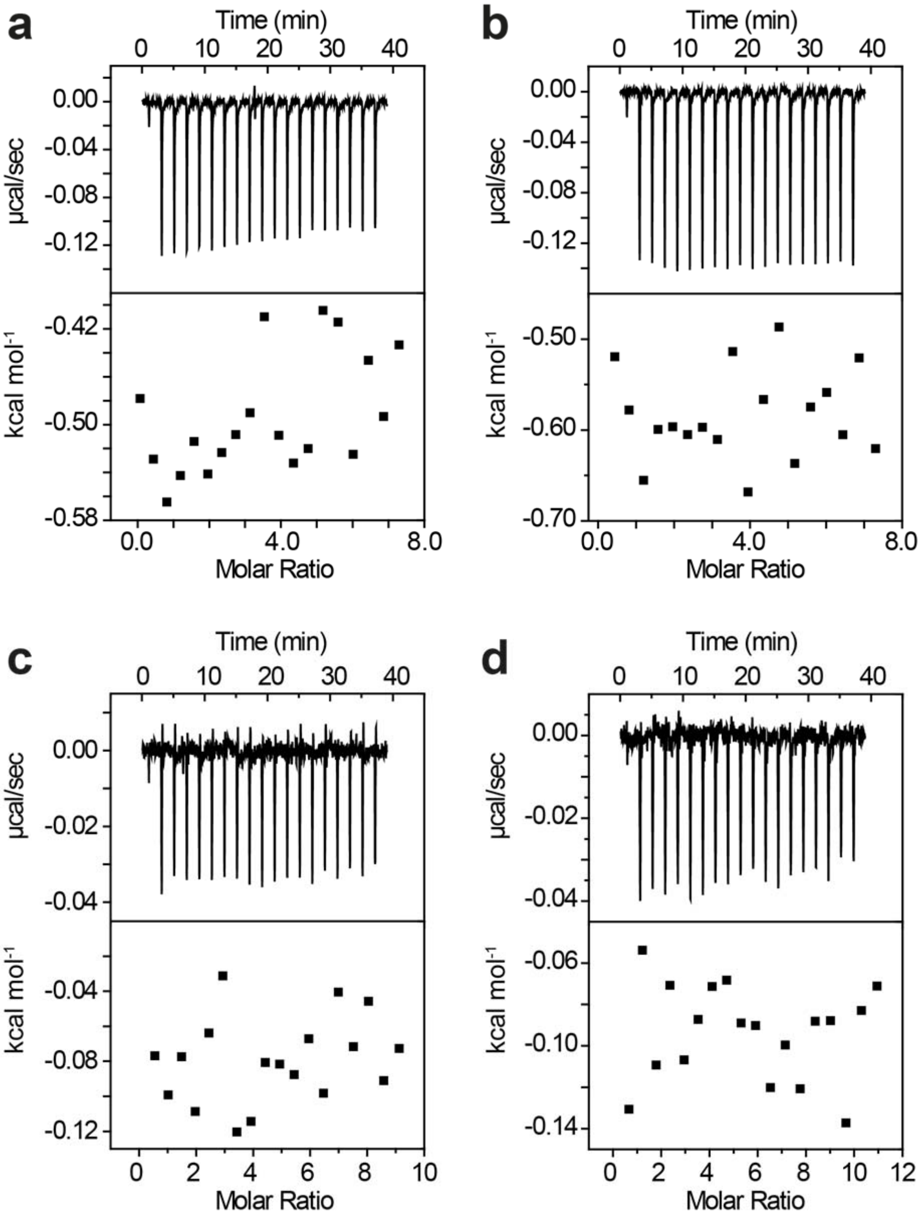

FIGURE S1. ITC experiments showing no high-affinity binding between TIL'E' and the a3 domain of FVIII with different sulfations: (*a*) FVIII a3 domain without any sulfation, FVIII $a3_{non\text{-}sulf}$, (*b*) FVIII a3 domain with Tyr1664 sulfated $a3_{sTyr1664}$, (*c*) FVIII a3 domain with Tyr1680 sulfated $a3_{sTyr1680}$, and (*d*) FVIII a3 domain with both Tyr1664 and Tyr1680 sulfated $a3_{sTyr1664\text{-}sTyr1680}$. Increasing the concentrations of FVIII a3 and TIL'E' in these experiments was not feasible due to solubility.

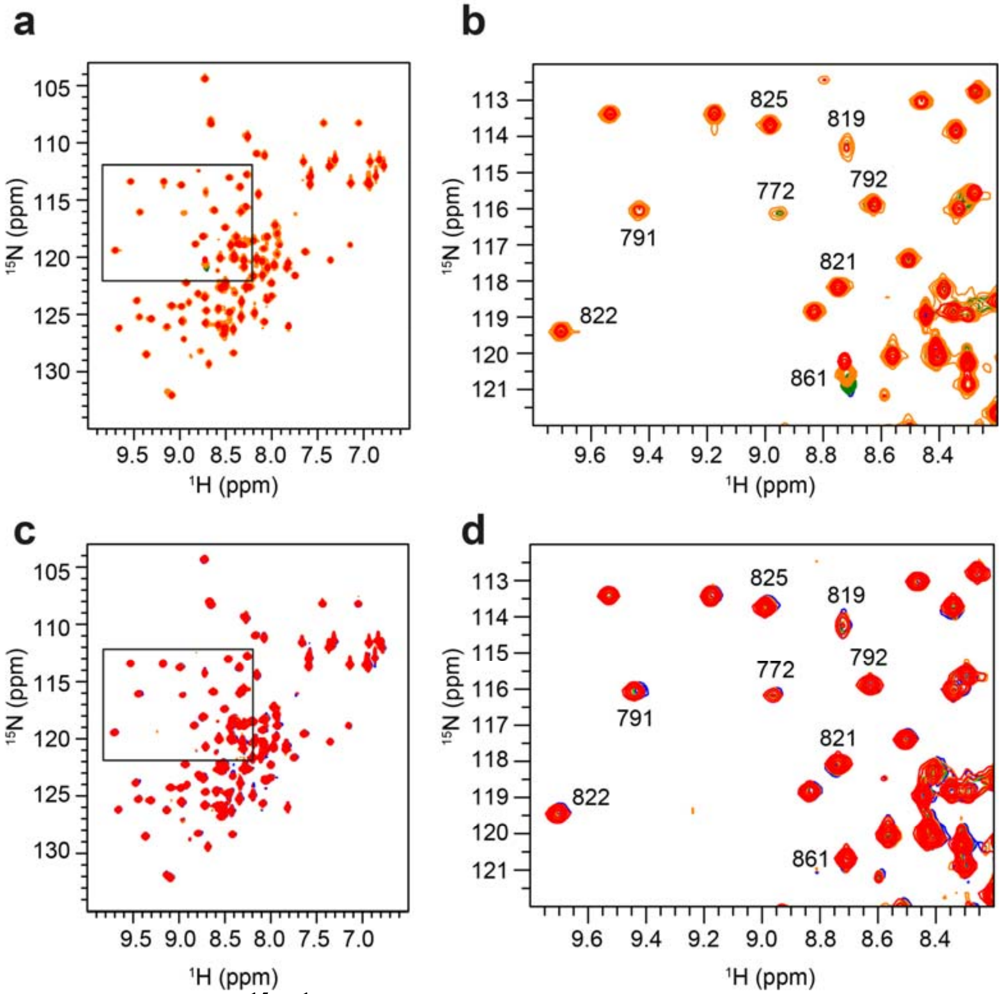

FIGURE S2. (*a*) and (*b*): ^{15}N-^{1}H spectrum of free TIL'E' at pH 6.5, 7.0, 7.5, and 8.0. (*c*) and (*d*): ^{15}N-^{1}H spectrum of free TIL'E' at NaCl concentrations of 100 mM, 150 mM, 200 mM, and 250 mM.

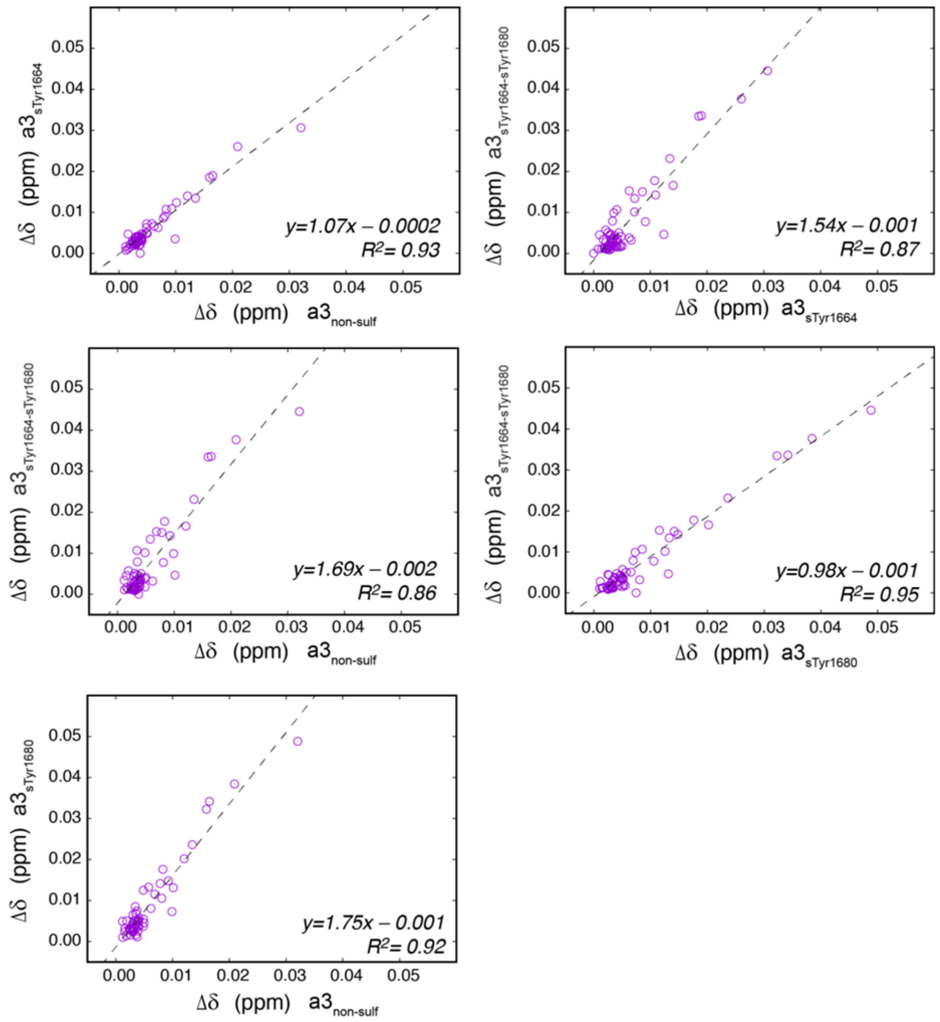

FIGURE S3. Comparison of ^{15}N-^{1}H TIL'E' chemical shift differences observed for titration of different FVIII a3 peptides with TIL'E'. The shift of His861 was excluded due to the strong pH dependence of this residue (see Fig. S2).

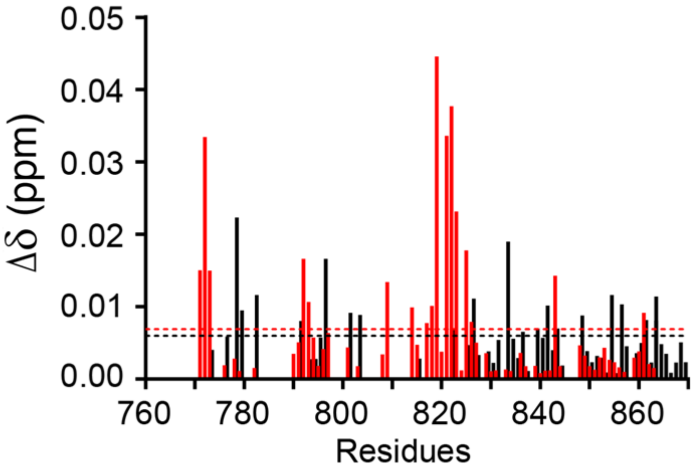

FIGURE S4. Lack of interaction between TIL'E' R816W and FVIII a3 peptide. The chemical shift differences, Δδ, observed in TIL'E' upon addition of $a3_{sTyr1664\text{-}sTyr1680}$ (red bars) compared to chemical shift differences of TIL'E' R816W upon addition of $a3_{sTyr1664\text{-}sTyr1680}$ (black bars). Whereas the binding between wild-type TIL'E' and $a3_{sTyr1664\text{-}sTyr1680}$ is specific and substantial, the addition of $a3_{sTyr1664\text{-}sTyr1680}$ to the type 2N mutant TIL'E' R816W leads only to minor and unspecific changes.

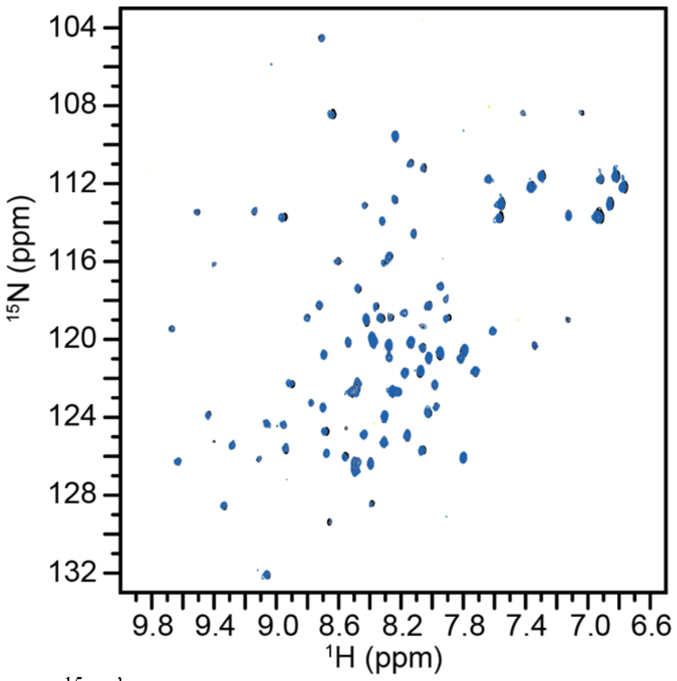

Figure S5. Overlay of ^{15}N-^{1}H correlation spectra of free TIL'E' (black) and TIL'E' with excess of FVIII and subsequent activation of FVIII with thrombin (blue). Many peaks disappear upon complex formation between TIL'E' and FVIII (Fig 2), however, upon activation of FVIII by thrombin and dissociation of the complex, the ^{15}N-^{1}H spectrum reverts to that of free TIL'E'. (see main text).

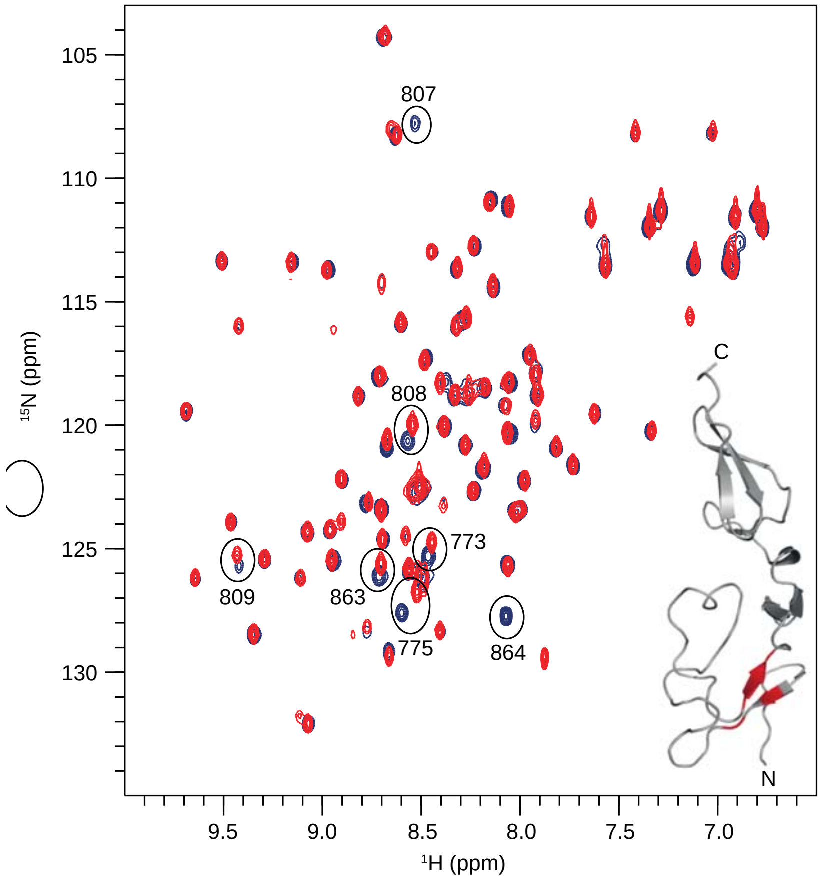

Figure S6. Overlay of ^{15}N-^{1}H correlation spectra of (red) TIL'E' with N-terminal perturbation (construct *iii*) and (blue) TIL'E' with native N-terminus. The good agreement between peak position and relative peak intensity of the two spectra show that the three-dimensional structures are very similar. Residues coloured red on the structure in the inset have $\Delta\delta > 0.04$ ppm (see main text).

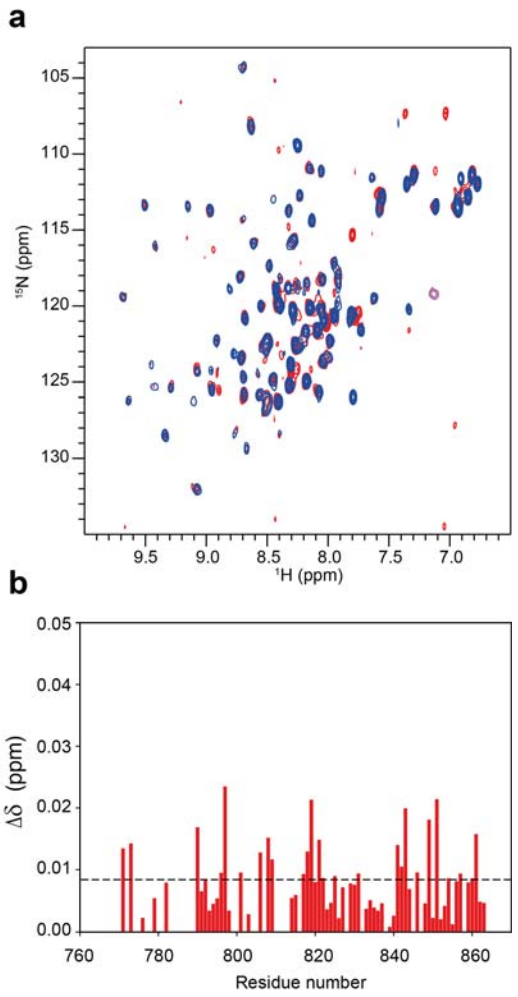

Figure S7. (*a*) Overlay of (blue) ^{15}N-^{1}H correlation spectrum of TIL'E' (construct *iii*) expressed in bacterial *E. coli.* cells and (red) natural abundance ^{15}N-^{1}H spectrum of TIL'E' (construct *iv*) expressed in mammalian HKB11 cells. (*b*) Chemical shift changes between 63 identified peaks of the two spectra in (*a*). The dashed line shows the average chemical shift changes. The good agreement between peak position and relative peak intensity of the two spectra show that correct folding and correct disulfide bridge formation is achieved in bacterial *E. coli*.
